# Parasite in Lepra

**Published:** 1881-07

**Authors:** 


### PARASITE IN LEPRA.

MM. Hillariet and Gaucher have communicated to the Société de Biologie the result of their preliminary experiments instituted for the purpose of ascertaining whether or not a parasite exists in the blood of leprous patients. After taking precautions to prevent the introduction of foreign fungi, these investigators discover the bacteria of leprosy to multiply and develop under their observation, giving rise to chains of articulated micrococci and to simple and ramifying filaments, presenting the ordinary appearance of the fungoid lower organism.—*Le Progrès Médical.*

